# Expression of SOCS1 and the downstream targets of its putative tumor suppressor functions in prostate cancer

**DOI:** 10.1186/s12885-017-3141-8

**Published:** 2017-02-24

**Authors:** Martin Chevrier, Diwakar Bobbala, Alberto Villalobos-Hernandez, Md Gulam Musawwir Khan, Sheela Ramanathan, Caroline Saucier, Gerardo Ferbeyre, Sameh Geha, Subburaj Ilangumaran

**Affiliations:** 1 Department of Pathology, Faculty of Medicine and Health Sciences, Université de Sherbrooke, Sherbrooke, Canada; 2 Department of Pediatrics, Immunology division, Faculty of Medicine and Health Sciences, Université de Sherbrooke, Sherbrooke, Canada; 3 Department of Anatomy and Cell Biology, Faculty of Medicine and Health Sciences, Université de Sherbrooke, Sherbrooke, Canada; 4 Department of Biochemistry, Faculty of Medicine, Université de Montréal, Montréal, Canada; 5 Centre de recherche du Centre hospitalier universitaire de Sherbrooke (CRCHUS), Sherbrooke, Québec J1H 5 N4 Canada

**Keywords:** Prostate cancer, SOCS1, Tumor suppressor, MET, p21

## Abstract

**Background:**

Suppressor of cytokine signaling 1 (SOCS1) is considered a tumor suppressor due to frequent epigenetic and micro-RNA-mediated repression of its gene expression in diverse cancers. In prostate cancer (PCa), elevated expression of miR-30d that targets SOCS1 mRNA is associated with increased risk of disease recurrence. SOCS1 can mediate its tumor suppressor functions by diverse mechanisms such as inhibiting the JAK-STAT signaling pathway, promoting the tumor suppressor functions of p53, attenuating MET receptor tyrosine kinase signaling and blocking the oncogenic potential of the cell cycle inhibitor p21^CIP1^ (p21). Here, we studied the expression of SOCS1 and the downstream targets of its putative tumor suppressor functions (p53, MET and p21) in human PCa specimens to evaluate their significance as markers of disease prognosis.

**Methods:**

Tissue microarrays were constructed of 78 archived prostatectomy specimens that were grouped according to the recommendations of the International Society of Urological Pathology (ISUP) based on the Gleason patterns. SOCS1, p53, MET and p21 protein expression were evaluated by immunohistochemical staining alongside the common prostate cancer-related markers Ki67, prostein and androgen receptor. Statistical correlations between the staining intensities of these markers and ISUP grade groups, local invasion or lymph node metastasis were evaluated.

**Results:**

SOCS1 showed diffuse staining in the prostatic epithelium. SOCS1 staining intensity correlated inversely with the ISUP grade groups (*ρ =* −0.4687, *p <*0.0001) and Ki67 (*ρ =* −0.2444, *p =* 0.031), and positively with prostein (*ρ =* 0.3511, *p =* 0.0016). Changes in SOCS1 levels did not significantly associate with those of p53, MET or p21. However, p21 positively correlated with androgen receptor expression (*ρ =* −0.1388, *p =* 0.0003). A subset of patients with regional lymph node metastasis, although small in number, showed reduced SOCS1 expression and increased expression of MET and p21.

**Conclusions:**

Our findings suggest that evaluating SOCS1 and p21 protein expression in prostatectomy specimens may have a prognostic value in identifying the aggressive disease. Hence, prospective studies with larger numbers of metastatic PCa specimens incorporating clinical correlates such as disease-free and overall survival are warranted.

**Electronic supplementary material:**

The online version of this article (doi:10.1186/s12885-017-3141-8) contains supplementary material, which is available to authorized users.

## Background

Prostate cancer (PCa) is the second most common cancer and fifth leading cause of cancer-associated mortality in men, with an estimated number of 1.1 million cases worldwide [1]. Despite early detection and treatment involving radical prostatectomy, radiotherapy and/or androgen deprivation, PCa continues to be a major cause of cancer-associated morbidity and mortality. Development of resistance to androgen deprivation therapy and acquisition of metastatic potential are the major causes of PCa mortality [2]. Cellular and animal models of PCa have elucidated many signaling pathways that render PCa refractory to treatment and contribute to metastatic dissemination [3, 4]. This knowledge can be exploited not only to develop personalized therapies for the recalcitrant disease but also to the development and testing of biomarkers for early detection of the aggressive disease.

The suppressor of cytokine signaling 1 (SOCS1) protein is considered a tumor suppressor because of frequent repression of the *SOCS1* gene promoter by CpG methylation in many types of cancers including hepatocellular carcinoma, leukemia and pancreatic adenocarcinoma [5–9]. SOCS1 expression is also inhibited by microRNAs such as miR-19 and miR-155 in human cancers [10–12]. *SOCS1* is one of the genes that are under-expressed in the androgen-independent PCa cell line LNCaP-C81 compared to the androgen-dependent LNCaP-33 cell line [13]. Even though methylation of the *SOCS1* promoter occurs only in 20% of PCa cases, increased expression of the SOCS1-targeting micro-RNA, miR-30d, has been reported to occur frequently in PCa [14, 15]. In fact, elevated miR-30d expression in PCa specimens correlates with early biochemical recurrence, supporting a tumor suppressor role for SOCS1 in PCa [15]. A number of studies have shown that SOCS1 attenuates growth of prostate cancer cells in vitro and in vivo [16–18].

The *SOCS1* gene is induced by diverse cytokines and growth factors, and inhibits their signaling in a negative feedback manner [8, 19]. SOCS1 has been shown to inhibit IL-6 and hepatocyte growth factor (HGF) signaling, which are implicated in PCa pathogenesis [16–18]. SOCS1 can exert its anti-tumor functions through diverse mechanisms. SOCS1 contains a central SH2 domain and C-terminal SOCS box. The SH2 domain binds to JAK kinases activated by cytokines and growth factor receptor tyrosine kinases (RTK), and thus blocks downstream signaling events [8, 19]. The SOCS box mediates ubiquitination of SOCS1-bound proteins, thereby promoting their degradation by proteasomes. We have shown that SOCS1 regulates HGF signaling by promoting ubiquitination and proteasomal degradation of the MET RTK [20, 21]. In cellular systems, SOCS1 co-operates with p53 to enforce oncogene-induced senescence [22]. However, in a mouse model of hepatocellular carcinoma, SOCS1 deficiency is associated with increased expression of a p53 target gene, the cyclin-dependent kinase inhibitor p21^CIP1/WAF1^ (p21) [23]. Even though p21 generally functions as a tumor suppressor, its cytosolic localization may promote tumor growth [24, 25]. Indeed, overexpression of p21 occurs in several human cancers and correlates with poor prognosis [24]. The relative contribution of the different downstream targets of SOCS1 in mediating tumor suppression in diverse cancers has not been studied yet. In PCa, even though p53 mutation is uncommon, MET and p21 are implicated in disease progression. Expression of MET occurs in 40% of localized PCa and correlates with Gleason score and lymph node metastasis, reaching nearly 100% in bone metastases [26–29]. Similarly, increased expression of p21 mRNA and protein in PCa has been associated with progression to androgen-independent cancer and resistance to apoptosis induction by chemotherapeutic agents [30, 31].

In this study, we investigated SOCS1 protein expression in prostatectomy specimens and its correlation to disease severity, with the goal of testing its utility as a prognostic biomarker. We examined the correlation between SOCS1 expression to those of its putative downstream targets of tumor suppression namely, p53, MET and p21 [21–23]. We observed significant inverse correlation between SOCS1 expression and disease severity. However, SOCS1 expression did not correlate with that of p53, MET or p21 in the whole study cohort. Within the study population, cases with regional lymph node metastasis, albeit small in number, showed decreased SOCS1 and increased MET and p21 expression.

## Methods

### Human prostatectomy specimens

From archived radical prostatectomy specimens received at the pathology department of the Centre hospitalier universitaire de Sherbrooke (CHUS), 175 consecutive samples collected between 2012 and 2015 were selected for this study. As the priority of this study was to evaluate tissue protein expression, and because archived specimens progressively lose antigenicity, only recent prostatectomy specimens collected between 2012 and 2015 were used. All the prostatectomy specimens were treatment-naïve. The study design also did not include serum PSA, disease outcome or patient follow-up, as only the recent prostatectomy specimens were prioritized to assess marker expression. Following approval by the Ethics committee of the Centre de Recherche du CHUS (Project #2014-734, 13–222), the patients or their families were contacted and consent was obtained from 90 patients. Among them, 12 cases were excluded due to a small tumor size of less than 5 mm in diameter, and 78 were included in this study.

### Tumor grading and classification

Tumor grading and staging data from the histopathology report were verified. Tumors were re-categorized according to the recommendations of the International Society of Urological Pathology (ISUP) into 5 groups, represented by the sum value of the first and second most prevalent Gleason patterns [32]: group 1 (Gleason score ≤6), group 2 (Gleason score 7 = 3 + 4), group 3 (Gleason score 7 = 4 + 3), group 4 (Gleason scores 8 = 4 + 4, 3 + 5, 5 + 3) and group 5 (Gleason score 9–10) [33]. All 78 cases harboured prostatic acinar adenocarcinoma. Among them, four showed foamy gland features, one displayed mucinous features and two others contained a ductal adenocarcinoma component. Within the study population, lymph node dissection had been performed in 36 cases, of which only 4 (N1) showed lymph node metastasis and the rest (N0, *n =* 32) did not. The specimens were categorized into two additional groups T2 and T3 stages, respectively representing those with carcinoma confined within the prostatic capsule (organ confined) and those that have breached this confinement (extra-prostatic extension) according to the American Joint Committee on Cancer (AJCC) Staging manual (7^th^ edition) [34].

### Construction of tissue microarray (TMA)

Following a thorough review of the haematoxylin and eosin (H&E)-stained slides, the largest tumor foci representative of the Gleason score was identified and correspondingly mapped on the paraffin block using a stereomicroscope (SZ51, Olympus) for precise targeting with the TMA MASTER system (3DHISTECH Ltd, Budapest, Hungary). The areas rich in tumor cells and representing the two prominent Gleason patterns were targeted. One large 2 mm diameter core was extracted from the mapped area and the 78 tumor cores were fitted in three TMAs using the TMA MASTER system. Cores containing clearly defined transitional epithelium, normal prostatic epithelium, low-grade and high-grade prostatic intraepithelial neoplasia (PIN) served as control or reference. PIN was defined according to WHO criteria [33].

### Immunohistochemistry

TMA sections of 4 μm thickness mounted on charged slides were deparaffinised, re-hydrated and heat-induced antigen retrieval was performed on DAKO PT Link pre-treatment module (Dako Pathology products, Mississauga, ON) using low or high pH Envision FLEX Target Retrieval Solutions (Dako). Subsequent steps were carried out on DAKO Autostainer Link. Endogenous peroxidase activity was blocked using Envision FLEX Peroxidase-blocking Reagent, followed by incubation with the primary antibodies (Ab) listed in Table 1. The EnVision FLEX /HRP (Dako) reagent containing a dextran polymer conjugated to anti-mouse Ig and anti-rabbit Ig and HRP was used as the secondary Ab. All washing steps were carried out using the Envision FLEX wash buffer and positive staining was detected by precipitation of diaminobenzidine (DAB) using the Envision FLEX DAB+ reagent. Slides were counterstained with hematoxylin, and coverslips were mounted using the Faramount mounting medium (Dako). The images were captured using an automated slide scanner (Aperio ScanScope XT, Aperio, Vista, California), analysed and scored in a blinded manner. A semi-objective scoring method (H-score) was used to quantify the staining. H-Score was evaluated on the whole tissue core. For the various markers, only the nuclear (Ki67, p53, AR and p21), the granular cytoplasmic (Prostein, SOCS1) or the membrane (MET) staining was considered for scoring. The staining intensities were graded on the scale of 0, 1, 2 and 3, representing absent, weak, moderate and strong staining. These values were multiplied by the respective percentage of staining to obtain the H-scores ranging from 0 to 300. Thus, the H-score recognises broad ranges of antigen expression and avoids arbitrary cut-offs.

**Table 1:** Antibodies used in this study

Ab	Source		Clone	Cat#	Dilution	Incubation time (min)	pH
AR	DAKO,	Mouse mAb	AR441	M3562	1/25	20	9.0
Ki67	DAKO	Mouse mAb	MIB-1	GA626	RTU	20	6.1
p21	DAKO,	Mouse mAb	SX118	M7202	1/50	20	9.0
p53	DAKO,	Mouse mAb	DO-7	GA616	RTU	20	9.0
Prostein	DAKO,	Mouse mAb	10E3	IR088	RTU	20	9.0
MET	CST	Rabbit mAb	D1C2	#8198	1/300	40	9.0
SOCS1	SCB	Rabbit polyclonal Ab	–	H-93	1/150	100	9.0
				(sc-9021)			

*RTU* Ready-To-Use dilution, *CST* Cell Signaling Technology, *SCB* Santa Cruz Biotechnology

### Statistical analysis

Statistical analyses were carried out using the GraphPad Prism 6 software (GraphPad Software, San Diego, USA). The data points were tested for Gaussian distribution before performing relevant statistical analyses, as indicated in each figure. For each dataset within groups, the distribution, mean and standard deviation within the 95% confidence limits are shown. Correlation between any two parameters was evaluated by non-parametric Spearman correlation test and the correlation coefficient (ρ) and the *p* value of the correlation are indicated within each figure. The slope was generated by non-linear regression curve-fit analysis. Non-parametric Kruskal-Wallis and Mann–Whitney tests were employed for comparing multiple or two groups, respectively. Statistically significant *p* values are represented by asterisks (* ≤ 0.05, ** ≤ 0.01), or the actual values are indicated within each figure.

## Results

### Ki67 and prostein expression in PCa correlates with tumor grade in the study cohort

**Fig. 1:**
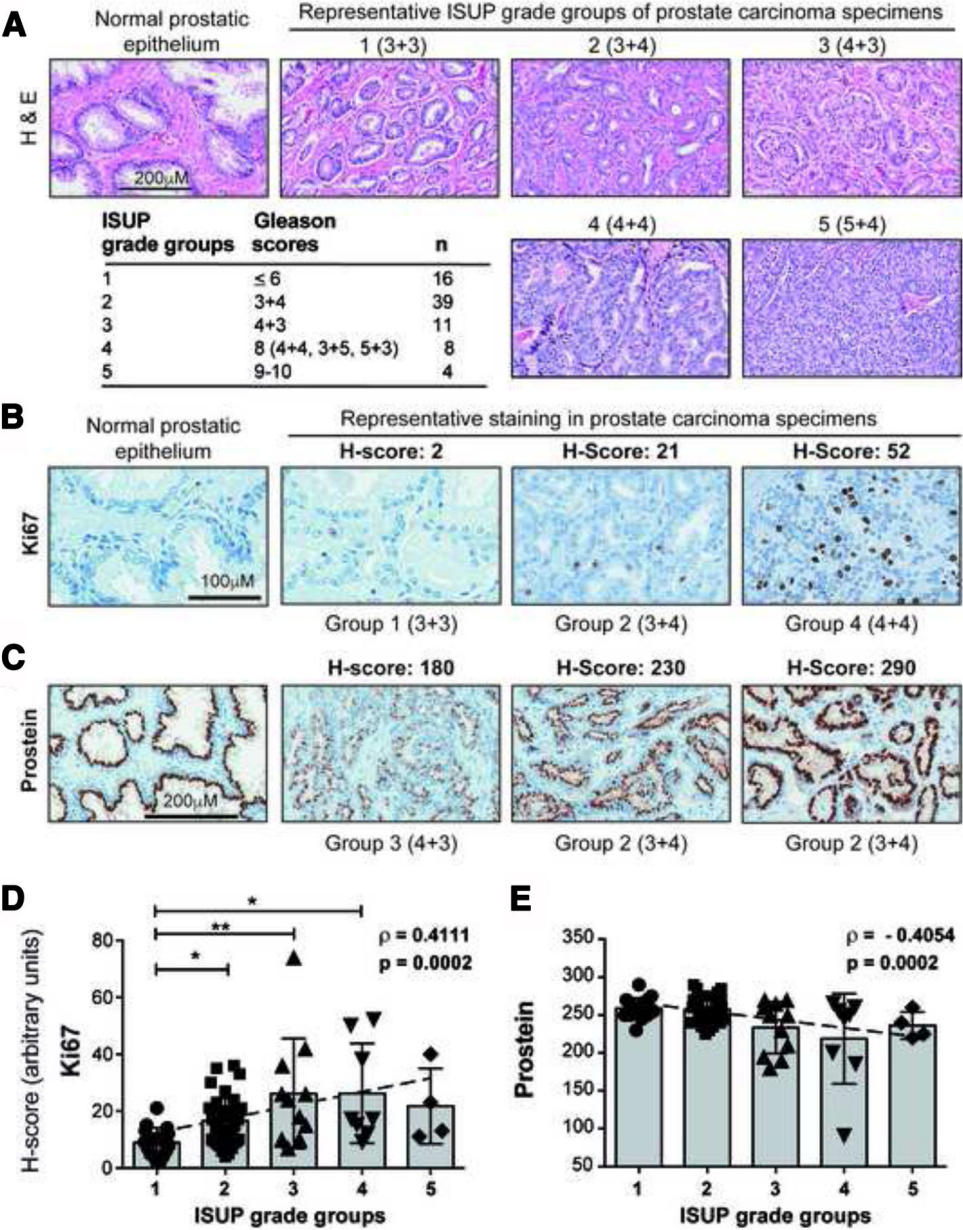
Ki67 and prostein staining in PCa specimens correlate with tumor grade*.* **a** Histopathological features of normal prostatic epithelium and representative ISUP grade groups of PCa specimens, as revealed by H&E staining. The Gleason scores corresponding to the ISUP grade group is given within parenthesis. **b**, **c** Representative immunohistochemical staining intensities of Ki67 and prostein in normal prostatic epithelium and in PCa specimens. The H-score of IHC staining intensity is indicated at the top of each specimen, and the ISUP grade groups with the Gleason scores in parenthesis at the bottom. Two specimens from group 2 are presented to illustrate the variation in staining intensity within groups. **d**, **e** Distribution of the H-scores of Ki67 (**d**) and prostein (**e**) across the ISUP grade groups. The slope represents the correlation between the H-score and the ISUP grade groups. Spearman’s rank correlation coefficient (ρ) and its statistical significance (*p*) are indicated. Statistical analyses between grade groups were carried out using one-way ANOVA adjusted with Kruskal-Wallis multiple comparisons test, and the significant *p* values are indicated

In order to evaluate the protein expression of SOCS1 and its putative downstream targets of tumor suppression in PCa, we constructed TMAs from archived PCa specimens and performed automated IHC staining to ensure staining homogeneity. To limit overlooking potential heterogeneity within individual tumor specimens, precautions were taken to include as much high-grade tumor foci as possible within the TMA core and not to exclude any smaller foci of higher grade. The TMAs were first stained by H&E to ascertain their histological features and distinguish carcinoma, PIN and benign structures, allowing their classification into five ISUP-recommended groups: 1 (*n =* 16), 2 (*n =* 39), 3 (*n =* 1), 4 (*n =* 8) and 5 (*n =* 4). The TMA sections were then stained for Ki67, a common cancer-associated marker (Fig. 1b). The high-grade tumors contained many Ki67+ cells compared to intermediate grade tumors, low grade tumors showed occasional Ki67+ cells, and the normal tissue rarely showed any positivity. Staining for prostein showed an opposite pattern compared to Ki67 staining. In general, low-grade tumors displayed intense prostein staining comparable to normal prostatic epithelium, and high-grade tumors showed reduced prostein staining (Fig. 1c). Consistent with these staining patterns, regression analysis showed highly significant positive correlation between the tumor grade and Ki67 staining, whereas prostein showed an inverse correlation (Fig. 1d and e). These results indicated that the study cohort was well represented by samples of different disease severity, despite containing relatively fewer high-grade tumors.

### SOCS1 staining decreases with disease severity

**Fig. 2:**
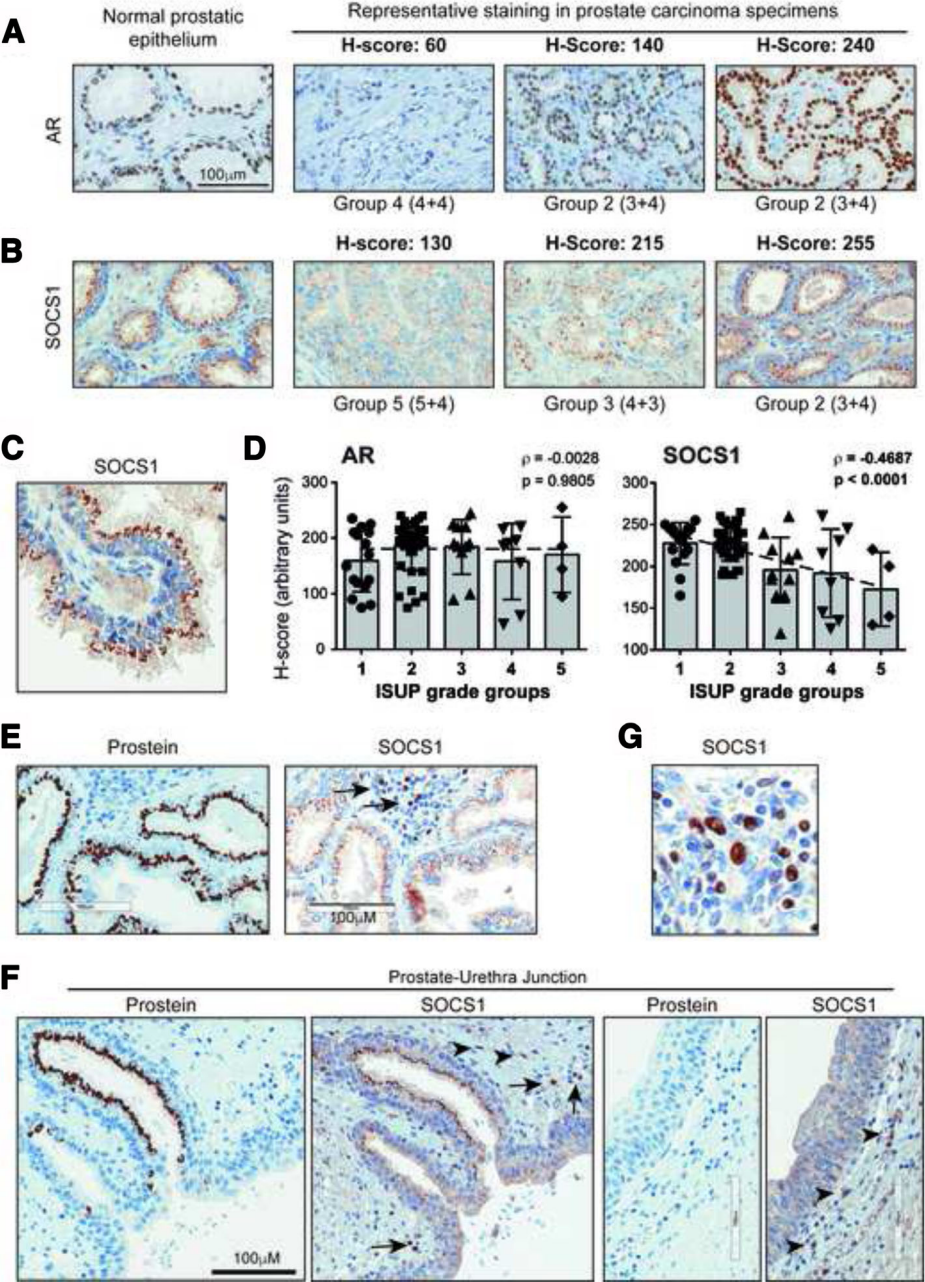
SOCS1 expression in PCa correlates inversely with the tumor grade. **a**, **b** Representative immunohistochemical staining intensities of androgen receptor (AR) and SOCS1 in normal prostatic epithelium and representative PCa specimens. The H-score of IHC staining intensity and the ISUP grade groups are indicated for each specimen. Two specimens from group 2 are presented to illustrate the variation in staining intensity within groups. **c** High power image of SOCS1 staining in normal prostatic epithelium. **d** Distribution of the H-scores of AR and SOCS1 across the ISUP grade groups. The slope shows the correlation between the H-score and the ISUP grade groups. Spearman’s rank correlation coefficient (ρ) and its statistical significance (*p*) are indicated. Statistical analyses between grade groups were carried out using one-way ANOVA adjusted with Kruskal-Wallis multiple comparisons test, and no significant differences were found. **e**-**g** Distinct staining patterns of prostein and SOCS1 in the prostate parenchyma (**e**) and at the prostate-urethra junction (**f**). Note the SOCS1 staining of adjacent immune cells (arrows) and stromal cells (arrowheads). **g** High power image of SOCS1 staining in lymphocytes within the prostatic stroma showing distinct nuclear positivity

It has been reported that androgen stimulation induces SOCS1 gene expression in PC3 cells [17]. Therefore, we evaluated the expression of androgen receptor (AR) and SOCS1 in PCa TMAs. The representative staining patterns and the corresponding tumor grades are shown in Fig. 2a and b. In contrast to antibodies specific to Ki67, prostein and AR that are approved for pathological diagnosis, the SOCS1 Ab is a research-grade rabbit polyclonal Ab from Santa Cruz Biotechnology (H-93, Catalogue #sc-9021). This SOCS1 Ab has been previously used on gastric and PCa specimens [17, 35] and by the Human Protein Atlas project [36]. This Ab detected a specific band of 27 kDa as expected in western blot analysis of COS-7 cells expressing human SOCS1 protein (Additional file 1: Figure S1). Two additional bands of higher MW estimated at 42 and 50 kDa were also observed only in SOCS1-transfected cells, suggesting that these bands might represent post-translationally modified SOCS1 protein. None of these bands were detected in control vector-transfected cells, indicating the specificity of the SOCS1 Ab used. We have optimized the staining conditions for IHC using this Ab, specifically pH of the antigen retrieval solution, primary Ab dilution and incubation time, using human fallopian tube specimens as documented in the manufacturer’s datasheet (data not shown). We obtained specific SOCS1 staining in PCa specimens as indicated by positive and negative cells within the same field of observation (Fig. 2b). We observed that SOCS1 showed a granular cytoplasmic staining of the prostatic epithelium with intense staining at the perinuclear region, predominantly on the luminal side (Fig. 2c). Whereas AR staining did not correlate with the ISUP grade groups, SOCS1 staining showed a significant negative correlation, similarly to prostein (Fig. 2d and Fig. 1e).

Although SOCS1 showed a staining pattern similar to that of prostein (Fig. 1c and Fig. 2b), it was unlikely that SOCS1 Ab displayed any cross-reactivity with prostein for the following reasons. While the prostein staining is highly specific to the prostatic epithelium and carcinoma as expected [37], the SOCS1 Ab also stained immune and stromal cells surrounding the gland and around the prostatic ducts (Fig. 2e and f). Moreover, in contrast to prostate epithelial cells, SOCS1 staining in immune cells showed a predominant nuclear distribution (Fig. 2e-g), indicating that SOCS1 staining is distinct from that of prostein.

**Fig. 3:**
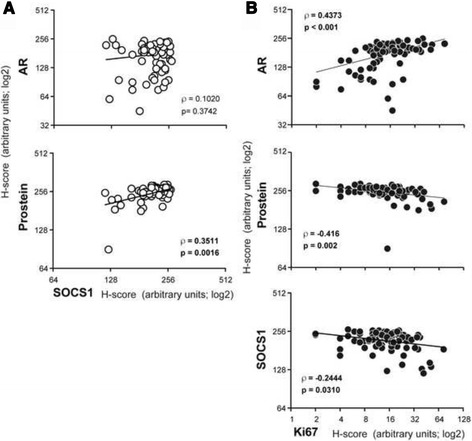
SOCS1 expression does not correlate with AR but correlates positively with prostein and negatively with Ki67 levels*.* **a** Correlation between the H-scores of SOCS1 and that of AR or prostein. **b** Correlation between the H-scores of Ki67 and that of AR, prostein or SOCS1. The slope represents the correlation between the H-scores. Spearman’s rank correlation coefficient (ρ) and its statistical significance (*p*) are indicated

Evaluation of the relationship between SOCS1 staining and that of AR did not show any association, whereas a positive correlation was observed between SOCS1 and prostein (Fig. 3a). However, AR staining correlated positively with Ki67 staining, whereas SOCS1 and prostein displayed significant negative correlation with Ki67 staining (Fig. 3b), suggesting that SOCS1 expression in PCa specimens might have a diagnostic significance.

### Relationship between SOCS1 expression and the putative downstream targets of its tumor suppressor functions

**Fig. 4:**
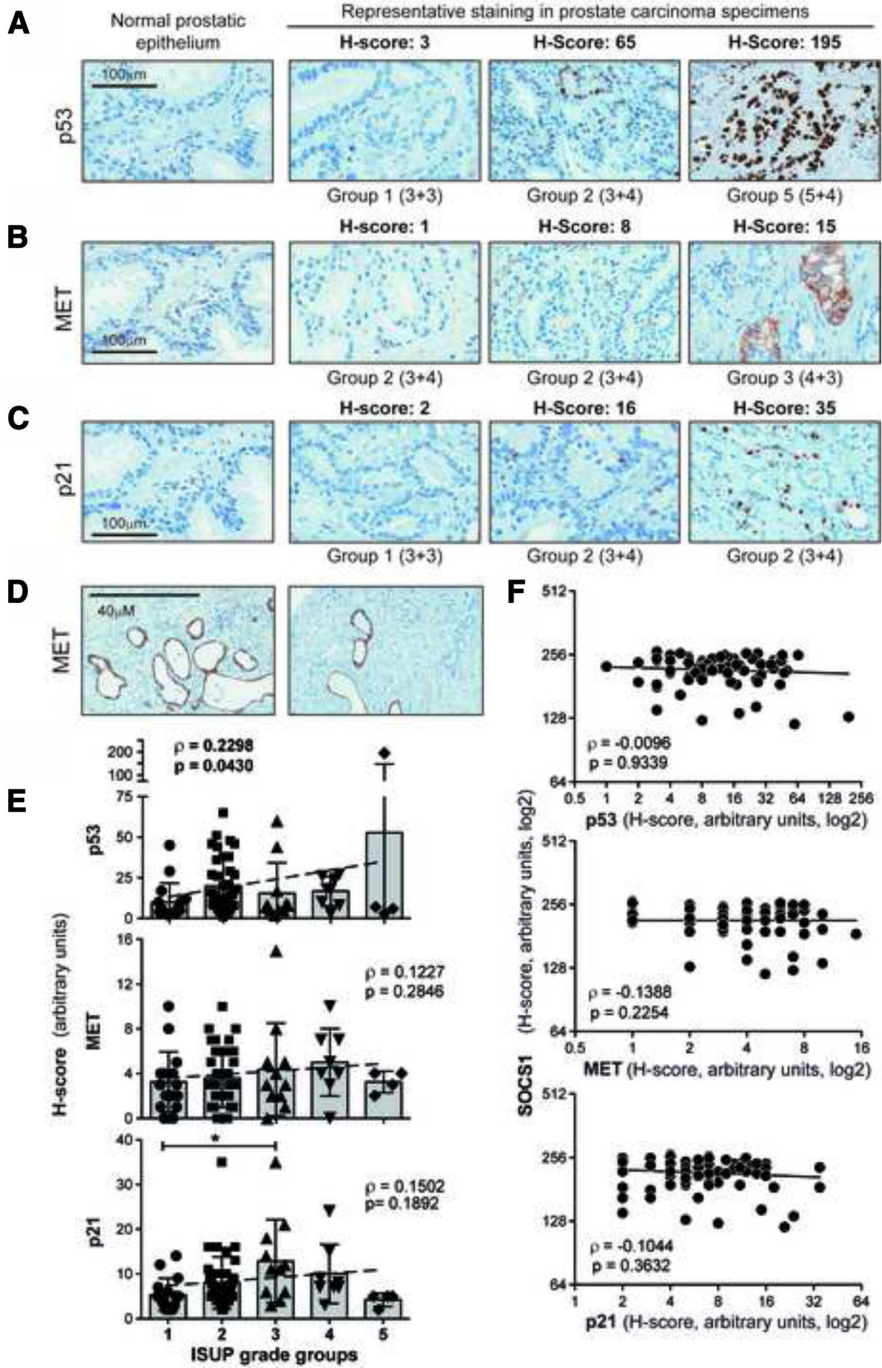
Expression of p53, MET or p21 does not correlate with SOCS1 staining in PCa*.* Representative immunohistochemical staining intensities of p53 (**a**), MET (**b**) and p21 (**c**) in normal prostatic epithelium and representative PCa specimens. The H-score of IHC staining intensity and the ISUP grade groups are indicated for each specimen. **d** Occasional staining of cyst-like structures by the anti-MET antibody. **e** Distribution of the H-scores of p53, MET and p21 across the ISUP grade groups. Slope represents the correlation between the H-score and the ISUP grade groups. Statistical analyses between grade groups were carried out using one-way ANOVA adjusted with Kruskal-Wallis multiple comparisons test, and the significant *p* values are indicated. **f** Correlation between the staining intensities of SOCS1 and that of p53, MET or p21. Spearman’s rank correlation coefficient (ρ) and its statistical significance (*p*) are indicated for E and F

SOCS1 can mediate tumor suppression by several potential mechanisms that were defined using cell and animal models. These include activation of p53-induced cellular senescence, and inhibition of oncogenic MET and p21 signalling through their ubiquitination and proteasomal degradation [20–23]. Hence, we postulated that the loss of SOCS1 might result in efficient p53 activation and compensatory upregulation of p53 expression, and increase in the expression of MET and p21. As each of these proteins is implicated in PCa pathogenesis [29, 30, 38], we evaluated their expression and correlation to SOCS1 levels in PCa TMAs. Representative expression patterns of p53, MET and p21 are shown in Fig. 4a-c. Occasionally, MET expression was confined to cyst-like structures and atrophic ducts with true membranous staining (Fig. 4d), although its significance is unclear. While p53 expression showed significant positive correlation with ISUP grade groups, MET and p21 only showed a tendency for positive correlation that were not statistically significant (Fig. 4e). SOCS1 staining did not correlate with the expression of p53, MET or p21 (Fig. 4f). These results suggested that the loss of SOCS1 might affect the downstream mediators of its tumor suppression mechanisms in a heterogeneous manner.

### Expression of p21 strongly correlates with AR levels

**Fig. 5:**
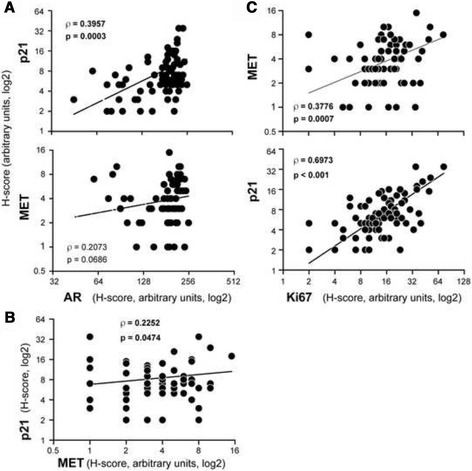
AR expression in PCa positively correlates with MET and p21 levels. Correlation between the staining intensities of (**a**) AR and p21 or MET, (**b**) MET and p21, and (**c**) Ki67 and MET or p21 in PCA specimens. Spearman’s rank correlation coefficient (ρ) and its statistical significance (*p*) are indicated

It has been reported that AR signalling represses MET expression, whereas androgen deprivation is associated with increased expression of p21 [30, 39], suggesting that escape to androgen independence would lead to increased expression of MET and p21, promoting their oncogenic functions. Therefore, we examined the relationship between the expression of AR and those of MET and p21. A strong positive association was observed between AR and p21 expression, whereas the positive correlation tendency between AR and MET was not statistically significant (Fig. 5a). MET and p21, which showed a significant positive correlation were also strongly associated with Ki67 staining (Fig. 5b and c). These results indicated that even though AR expression did not correlate with the ISUP grade or MET (Fig. 2d), AR and MET signalling might influence the expression of p21 expression and its oncogenic functions.

### Expression of SOCS1 and its downstream targets in locally-advanced and metastatic PCa

**Fig. 6:**
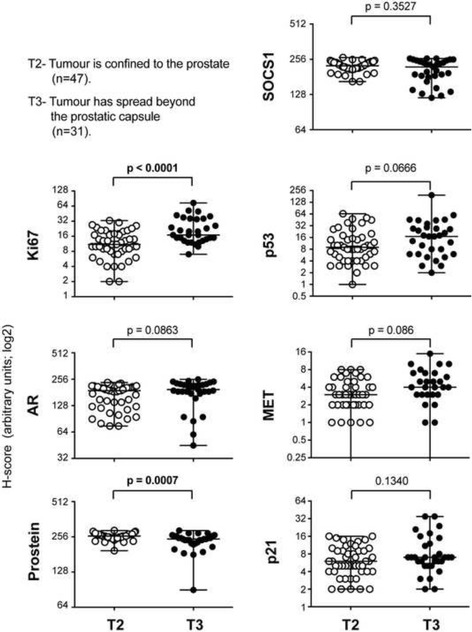
SOCS1 expression is not significantly different between organ-confined PCa and tumors with extra-prostatic extension. Tumors confined within the prostatic capsule (T2, *n =* 47) and those that have spread beyond (T3, *n =* 31) were compared for the staining intensities of Ki67, AR, prostein, SOCS1, p53, MET or p21. Median values with range (bars) are indicated. Nine samples that showed no MET expression (5 in T2, 4 in T3) were not indicated due to log scale of the y-axis. The datasets were compared by Mann–Whitney test and statistically significant *p* values are indicated in bold

**Fig. 7:**
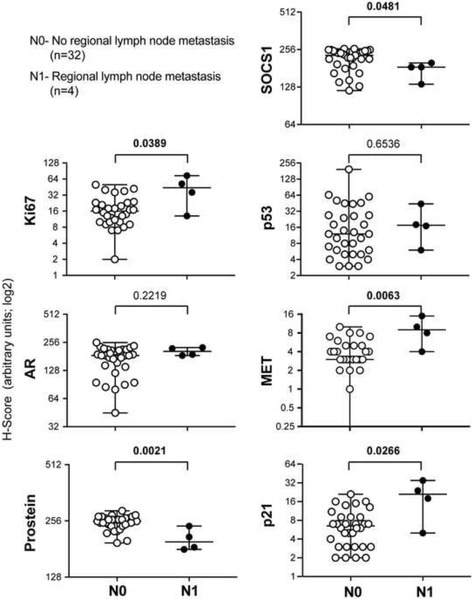
Decreased expression of SOCS1 and increased expression of MET and p21 in PCa are associated with regional lymph node spread. Tumors from PCa patients without lymph nodes metastasis (N0, *n =* 32) were compared to those with lymph node metastasis (N1, *n =* 4) for the staining intensities of Ki67, AR, prostein, SOCS1, p53, MET or p21. Median values with range (bars) are indicated. The datasets were compared by Mann–Whitney test. Statistically significant *p* values are indicated in bold

We grouped the specimens into tumors that are either confined within (T2, *n =* 47) or spread beyond (T3, *n =* 41) the prostatic capsule. Evaluation of the marker expression in these two groups revealed that the invasive tumors showed significantly higher Ki67 and lower prostein staining (Fig. 6). On the other hand, expression of AR, SOCS1, p53, MET or p21 was not significantly different between T2 and T3 groups. A subset of the samples, for which tumor spread to the regional lymph node had been evaluated (*n =* 36), were grouped into N0 (*n =* 32) and N1 (*n =* 4) subgroups, respectively representing cases without or with metastatic spread to the regional lymph node. Even though the latter group contained only a limited number of cases, their Ki67 expression was significantly higher and prostein levels were significantly lower compared to specimens in the former group, while the AR expression was comparable (Fig. 7). Moreover, specimens from cases with lymph node metastasis showed significantly reduced SOCS1 staining and concomitant increase in MET and p21 expression. Although these findings need to be confirmed in larger study populations, they suggest that SOCS1, MET and p21 levels could be useful as predictive markers of metastatic PCa.

## Discussion

Given that the expression of SOCS1 is regulated at post-transcriptional level by microRNAs [10–12, 15], detection of SOCS1 protein in cancer tissues would represent a direct approach to evaluate SOCS1 as a potential cancer biomarker. However, detection of endogenous SOCS1 protein has been particularly difficult for two reasons. Firstly, the basal *SOCS1* gene expression in normal tissues is very low; however, it is induced by myriad of inflammatory cytokines, growth factors, chemokines and other mediators such as prostaglandins and androgens [17, 40–42]. Secondly, most available SOCS1 antibodies are not sensitive enough to detect the endogenous SOCS1 protein. Hence, the literature on SOCS1 expression is mostly restricted to quantification of *SOCS1* mRNA with only a few reports on SOCS1 protein expression [9, 17, 35, 43, 44]. Given that *SOCS1* gene repression occurs in many cancers by diverse mechanisms, clearly there is a need for developing and testing more sensitive and specific SOCS1 antibodies for diagnostic purpose in surgical pathology.

We observed different staining intensities of SOCS1 in prostatectomy specimens that showed strong inverse correlation with the ISUP grade groups and Ki67 staining. Moreover, tumors with regional lymph node involvement showed significantly lower SOCS1 expression compared to those without. These findings indicate that SOCS1 expression is diminished during PCa progression and support the potential tumor suppressor role of SOCS1 in this cancer. Moreover, the finding that SOCS1 staining was reduced but not abolished in PCa specimens is consistent with the earlier reports that repression by CpG methylation occurs only in a small subset of PCa, whereas the expression of the SOCS1-targeting miR-30d is more frequent [14, 15]. Hence, evaluating SOCS1 protein would be more informative than quantifying CpG methylation of the *SOCS1* gene, *SOCS1*-targeting micro-RNA or *SOCS1* mRNA levels.

In the current study, we examined how reduced expression of SOCS1 protein in PCa specimens impacted on p53, MET and p21, which are regulated by SOCS1 [21–23] and are implicated in PCa progression [29, 30, 38]. In the PCa grade groups, we observed variable protein staining for p53, MET and p21, of which only p53 correlated with either disease severity, and none of them correlated with SOCS1protein expression. Evaluation of p53 in human cancers has restricted diagnostic/prognostic utility because increased expression generally correlates with mutated or dysfunctional p53 [45]. Nonetheless, we postulated based on the requirement of SOCS1 to mediate p53-dependent cellular senescence [22] that SOCS1 deficiency might increase p53 expression, similarly to inactivating p53 mutations. Likewise, we expected increased protein levels of MET and p21 in cases where SOCS1 was limiting, as the latter attenuates MET signalling and p21 expression at least partly via ubiquitination and proteasomal degradation [21, 23]. Even though there was a tendency of inverse correlation between SOCS1 and p53, MET or p21, they were not statistically significant (Fig. 4f). Clearly, studies on larger cohorts are needed either to confirm this tendency, or to support the alternate possibility that the decrease in SOCS1 expression need not necessarily affect all of its downstream mediators of tumor suppression, as the SOCS1-mediated suppression pathways may vary in individual cancers. Besides, other molecules/pathways might influence the downstream mediators of SOCS1 to a variable extent in individual cancers.

Major prognostic determinants of PCa are grade, local extension, lymphovascular invasion and lymph node metastasis. Still, many intermediate grade carcinomas extend outside the prostate gland or metastasize, and many high-grade carcinomas are organ-confined at time of surgery. In such situations, additional prognosis markers would be important in determining whether more aggressive treatment is warranted. A lot of effort has been made in the last two decades to develop and refine diagnostic and prognostic PCa biomarkers [46]. The former group (e.g., % of free PSA over total PSA, PCA3/DD3 gene expression) strives to achieve higher specificity than total serum PSA levels in order to overcome false-positive results and avoid unnecessary biopsies. On the other hand, prognostic biomarkers such as early PCa antigen-2 (EPCA-2) and post-operative PSA velocity (PSAV) and doubling time (PSAD) have shown promise in predicting PCa aggressiveness, risk of non-organ confined disease and disease recurrence. SOCS1 immunostaining may complement the latter group in predicting aggressive disease, although further studies in larger cohorts with non-organ confined disease and metastatic PCa as well as development of refined reagents with improved sensitivity and specificity are needed.

Androgen receptor (AR) signalling is a major oncogenic driver in PCa progression via induction of promitotic genes, and the AR gene is often amplified or mutated in PCa that have become resistant to androgen deprivation therapy [47]. PCa with acquired resistance to therapy display distinct AR-responsive gene expression signature [48]. In recent studies, the AR splice variant AR-V7 has emerged as a predictive biomarker of PCa responsiveness to next generation androgen targeting therapies [49]. We observed that AR expression did not vary significantly across the PCa grades or the tumor stages T2 and T3. However, a strong positive correlation was observed between AR expression and Ki67 staining, in agreement with the ability of AR signalling to stimulate cell proliferation [47]. Even though androgen stimulation induces *SOCS1* gene expression in PC3 cells [17], we did not find significant correlation between AR and SOCS1 protein expression. Nonetheless, SOCS1 protein level showed significant negative correlation with Ki67 staining, reflecting the loss of SOCS1-dependent control of other growth stimulatory pathways.

Tumors with regional lymph node metastasis, despite being limited in number, showed significantly lower SOCS1 and prostein expression and higher levels of Ki67, MET and p21 than those without lymph node involvement (Fig. 7). SOCS1 might impinge on multiple signaling pathways that promote aggressive growth, migration, invasion and metastasis, resulting in metastatic spread to regional lymph nodes. Increased MET expression had been reported in metastatic PCa [28]. We have recently shown using PCa cell lines that SOCS1 inhibits HGF-induced MET signaling and attenuated their migration and invasion [18]. Consistent with this report, MET overexpression correlated with high proliferation and regional lymph node metastasis (Fig. 5 and Fig. 7). However, we did not observe significant correlation between SOCS1 and MET protein expression. Several factors could contribute to this discrepancy between the studies on cell lines and in primary cancers. For instance, in primary cancers retaining SOCS1 expression, MET mutations might allow escape from SOCS1-mediated regulation. Alternatively, signaling pathways other than MET might contribute to aggressive growth of cancer cells retaining SOCS1 expression. The association between p21 and metastasis, but not with tumor grade is also intriguing. Although the number of metastatic cancers included in our study is small, our finding raises the possibility that the proposed function of cytosolic p21 in promoting cell motility [50] might contribute to PCa metastasis.

Even though the loss of SOCS1 did not correlate with increased MET or p21 expression as we had expected, our findings support the tumor suppressor function of SOCS1, and the oncogenic potential of MET and p21 in PCa. The expression of p21 also showed a strong correlation to AR levels, which is expressed in androgen-independent PCa and contributes to disease progression in a ligand-independent manner, renewing the interest in AR blockade [51, 52]. MET and AR levels also showed a marked tendency for positive correlation, although this was not statistically significant. Overall, the expression of SOCS1, MET and p21 in PCa specimens may be useful to identify cases that are prone to metastatic spread and thus to put them on more aggressive treatment and/or under close monitoring. While highly specific, clinical grade anti-p21 Abs are available from DAKO, anti-MET Abs suitable for such use are still in development [53] and efforts must be made to develop clinical grade SOCS1 Abs.

Our study design did not include patient follow-up for responsiveness to therapy or survival. Therefore, we analyzed correlation between *SOCS1* gene expression and patient survival in datasets obtained from the cBioportal (http://www.cbioportal.org/) [54] and PrognoScan (http://www.abren.net/PrognoScan/) [55] cancer web portals. We did not find any significant correlation between *SOCS1* gene expression and patient survival in both datasets (Additional file 2: Figure S2). The currently available public cancer databases do not contain protein expression data. Therefore, it is necessary to carry out prospective studies on SOCS1 protein expression in PCa and other cancers with a wider scope, including treatment responsiveness, disease-free survival and overall survival. The present study lays foundation for such future investigations.

## Conclusions

Collectively, our findings indicate that (i) evaluating SOCS1 protein expression may have a prognostic significance in PCa, however this requires development of highly specific and sensitive clinical grade antibodies, and (ii) MET and p21, which are partly regulated by SOCS1, may also be useful in identifying aggressive cancers. Clearly, prospective studies in larger cohorts are needed to evaluate these parameters as well as their correlation to resistance to androgen ablation therapy, serum biomarkers and distant metastasis in order to validate their utility as prognostic markers and, possibly as targets for personalized therapy.

## Additional files

Additional file 1: Figure S1. Specificity of the SOCS1 Ab. (PDF 569 kb)
Additional file 2: Figure S2. Lack of correlation between *SOCS1* gene expression in PCa specimens and patient survival. (PDF 201 kb)

## Abbreviations

Ab: Antibody
AR: Androgen receptor
HGF: Hepatocyte growth factor
ISUP: International society of urological pathology
p21: p21^CIP1/WAF1^
PCa: Prostate cancer
RTK: Receptor tyrosine kinase
SOCS1: Suppressor of cytokine signaling 1
TMA: Tissue microarray
