# Supplementary material for: Expression of SOCS1 and the downstream targets of its putative tumor suppressor functions in prostate cancer
Source: BMC Cancer. 2017 Feb 24;17:157. doi: 10.1186/s12885-017-3141-8 (PMC5326496; doi:10.1186/s12885-017-3141-8)
Supplement: Additional file 1: Figure S1. — Specificity of the SOCS1 Ab. (PDF 569 kb) [file 12885_2017_3141_MOESM1_ESM.pdf]

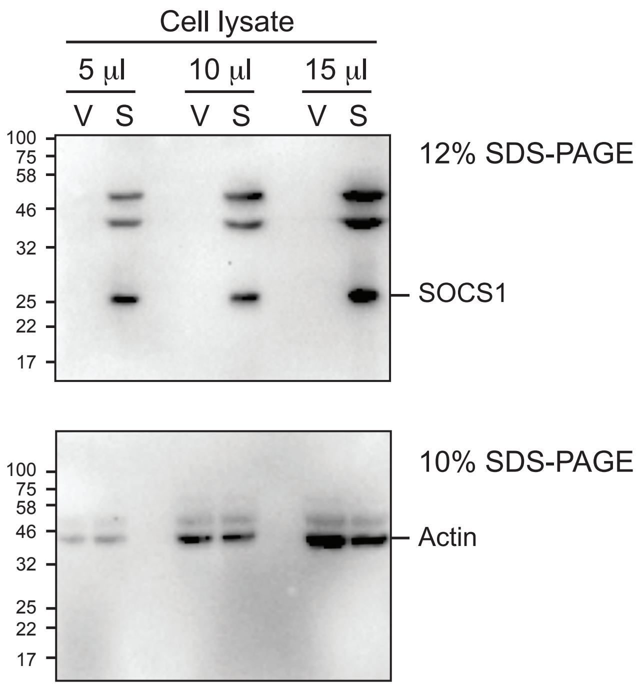

Supplementary Figure 1. Specificity of the SOCS1 Ab. Cos-7 cells cultured in 60mm Petri dishes were transfected with human SOCS1 construct (S) or the control vector pcDNA 3.0 (V). The transfected cells were lysed 36h later in 100 µl SDS-PAGE sample buffer, and the indicated quantities of whole cell lysates were analyzed by western blot using the H-93 anti-SOCS1 Ab from Santa Cruz Biotechnology at 1:500 dilution. A duplicate blot was probed for actin (Ab from Sigma) that served as loading control for vector-transfected cells.
