# Supplementary material for: Expression of SOCS1 and the downstream targets of its putative tumor suppressor functions in prostate cancer
Source: BMC Cancer. 2017 Feb 24;17:157. doi: 10.1186/s12885-017-3141-8 (PMC5326496; doi:10.1186/s12885-017-3141-8)
Supplement: Additional file 2: Figure S2. — Lack of correlation between SOCS1 gene expression in PCa specimens and patient survival. (PDF 201 kb) [file 12885_2017_3141_MOESM2_ESM.pdf]

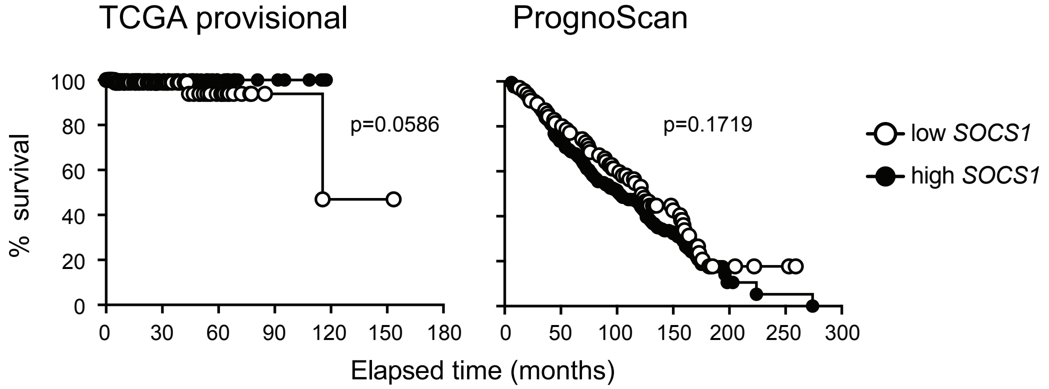

Supplementary Figure 2. Lack of correlation between *SOCS1* gene expression in PCa specimens and patient survival.

Data on PCa were mined from the cBioportal (http://www.cbioportal.org/) [1, 2] and PrognoScan (http://www.abren.net/PrognoScan/) [3] cancer web portals, and analyzed for the correlation between *SOCS1* gene expression and patient survival. From the 497 PCa datasets from The Cancer Genome Atlas (TCGA) provisional database accessed through the cBioportal, 248 cases that represented top high and bottom low 25 percentile of *SOCS1* mRNA expression (RNA Seq V2 RSEM) were selected for survival analysis. Out of the 281 PCa patients in Prognoscan database, 211 expressed higher and 70 expressed lower levels of *SOCS1* mRNA. Overall survival time was calculated from the date of diagnosis until the date of death or the last follow-up examination. Kaplan-Meier survival curves of patients with high or low *SOCS1* mRNA expression were compared using the Log-rank (Mantel-Cox) test, and no significant difference was observed between the two groups in both datasets. Given that SOCS1 expression in PCa is regulated at the post-transcriptional level by micro-RNA [4], and that immune cells in tumor stroma are likely to express the *SOCS1* gene, the RNA seq data of PCa datasets may not truly reflect SOCS1 protein expression. Therefore, future studies designed to evaluate SOCS1 protein expression in PCa specimens by IHC along with correlation to clinical parameters are clearly needed.

References:

1. Gao J, Aksoy BA, Dogrusoz U, Dresdner G, Gross B, Sumer SO, Sun Y, Jacobsen A, Sinha R, Larsson E et al: Integrative analysis of complex cancer genomics and clinical profiles using the cBioPortal. Sci Signal 2013, 6(269):pl1.

2. Cerami E, Gao J, Dogrusoz U, Gross BE, Sumer SO, Aksoy BA, Jacobsen A, Byrne CJ, Heuer ML, Larsson E et al: The cBio cancer genomics portal: an open platform for exploring multidimensional cancer genomics data. Cancer Discov 2012, 2(5):401-404.

3. Mizuno H, Kitada K, Nakai K, Sarai A: PrognoScan: a new database for meta-analysis of the prognostic value of genes. BMC Med Genomics 2009, 2:18.

4. Kobayashi N, Uemura H, Nagahama K, Okudela K, Furuya M, Ino Y, Ito Y, Hirano H, Inayama Y, Aoki I et al: Identification of miR-30d as a novel prognostic maker of prostate cancer. Oncotarget 2012, 3(11):1455-1471.
